# Endoskopische Vermessung von Nasenseptumdefekten

**DOI:** 10.1007/s00106-021-01101-5

**Published:** 2021-09-03

**Authors:** Jean-Claude Rosenthal, Eric L. Wisotzky, Carsten Matuschek, Melanie Hobl, Anna Hilsmann, Peter Eisert, Florian C. Uecker

**Affiliations:** 1 Vision and Imaging Technologies, Fraunhofer Heinrich-Hertz-Institut HHI, Einsteinufer 37, 10587 Berlin, Deutschland; 2 MKG-Klinik, Charité – Universitätsmedizin Berlin, Berlin, Deutschland; 3 Visual Computing, Humboldt Universität zu Berlin, Berlin, Deutschland; 4 HNO-Klinik, Charité – Universitätsmedizin Berlin, Berlin, Deutschland

**Keywords:** Endoskopie, Nasenerkrankungen, Bildgesteuerte Therapie, Rekonstruktive chirurgische Verfahren, 3D-Rekonstruktion, Endoscopy, Nose diseases, Image-guided therapy, Reconstructive surgical procedures, 3D reconstruction

## Abstract

**Hintergrund:**

Die vielfältigen unangenehmen Symptome von Nasenseptumdefekten (NSD) führen zu einer deutlichen Einschränkung der Lebensqualität. NSD können mittels patientenspezifischer Implantate oder durch eine Op. verschlossen werden. Implantate werden dabei durch Silikonabformungen unter Vollnarkose, ggf. in lokaler Betäubung, oder anhand von 3D-Modellen aus computertomographischen (CT-)Daten erstellt. Nachteile für die Patientensicherheit sind ein erhöhtes Morbiditätsrisiko oder die Strahlenbelastung.

**Methodik:**

Für die Hals‑, Nasen- und Ohren-(HNO)-Chirurgie wird ein schonender Ansatz zur Behandlung von NSD mit einer neuen bildbasierten, kontakt- und strahlungsfreien Messmethode unter Nutzung eines Stereoendoskops vorgestellt. Das Verfahren beruht ausschließlich auf Bilddaten und nutzt echtzeitfähige Bildverarbeitungsalgorithmen zur Berechnung von 3D-Informationen. Es ist beliebig oft wiederholbar und wurde bereits erfolgreich in der robotergestützten Chirurgie und in der OP-Mikroskopie eingesetzt. Daher wurde diese Methode für die Nasenchirurgie erweitert, für die es zusätzliche anatomische und stereoskopische Herausforderungen gibt.

**Ergebnisse:**

Nach Auswertung von 3 relevanten Messgrößen (NSD-Ausdehnung: axial, koronal und Umfang) von 6 Patienten und Vergleich der Ergebnisse von 2 Stereoendoskopen mit vorhandenen CT-Daten zeigte sich: Die bildbasierten Messergebnisse können vergleichbare Genauigkeiten wie CT-Daten erzielen. Bei einem Patienten wurden die Daten nur teilweise ausgewertet, da der NSD größer als das endoskopische Sichtfeld war.

**Schlussfolgerung:**

Aufbauend auf den sehr guten Messwerten wird ein Therapieverfahren skizziert, welches die Herstellung von patientenspezifischen NSD-Implantaten auf Basis endoskopischer Daten ermöglicht.

## Abstract (en)

**Background:**

Nasal septum perforations (NSP) have many uncomfortable symptoms for the patient and a highly negative impact on quality of life. NSPs are closed using patient-specific implants or surgery. Implants are created either under anesthesia using silicone impressions or using 3D models from CT data. Disadvantages for patient safety are the increased risk of morbidity or radiation exposure.

**Materials and methods:**

In the context of otorhinolaryngologic surgery, we present a gentle approach to treating NSP with a new image-based, contactless, and radiation-free measurement method using a 3D endoscope. The method relies on image information only and makes use of real-time capable computer vision algorithms to compute 3D information. This endoscopic method can be repeated as often as desired in the clinical course and has already proven its accuracy and robustness for robotic-assisted surgery (RAS) and surgical microscopy. We expand our method for nasal surgery, as there are additional spatial and stereoperspective challenges.

**Results:**

After measuring 3 relevant parameters (NSP extension: axial, coronal, and NSP circumference) of 6 patients and comparing the results of 2 stereoendoscopes with CT data, it was shown that the image-based measurements can achieve comparable accuracies to CT data. One patient could be only partially evaluated because the NSP was larger than the endoscopic field of view.

**Conclusion:**

Based on the very good measurements, we outline a therapeutic procedure which should enable the production of patient-specific NSP implants based on endoscopic data only.

In der HNO-Chirurgie und speziell in der Rhinologie ist es von großer Bedeutung, dass die Nasenhöhle und deren Belüftungssystem störungsfrei und funktionsfähig sind. Eine Störung des Luftstroms stellt eine hohe Beeinträchtigung der Lebensqualität des Patienten dar. Dies trifft insbesondere für das Krankheitsbild der Nasenseptumdefekte (NSD) zu.

Bei einem Nasenseptumdefekt (NSD) wird die Atmung durch den Kontinuitätsdefekt im knorpeligen oder knöchernen Anteil der Nasenscheidewand beeinträchtigt, wobei die mukoperichondrale oder mukoperiostale Auskleidung fehlt. In Abb. 1a sind die anatomischen Details an einem Nasspräparat im Querschnitt und ein Überblick über die Lage der Nasenscheidewand erfasst. Die Abb. 1b zeigt eine Perforation unter endoskopischer Sicht.

**Figure 1:**
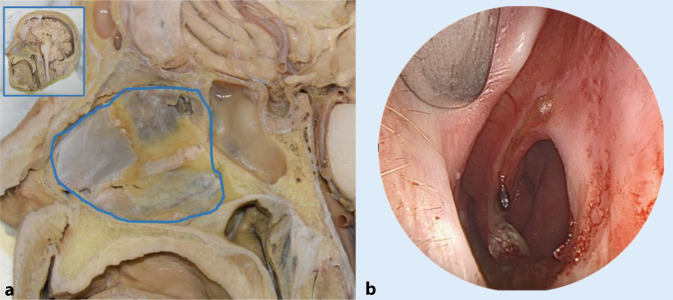

Ein NSD kann zu vielfältigen und unangenehmen Symptomen führen, wie eingeschränkte Atmung, Nasenbluten, trockene bzw. reflektorisch zu feuchte Nase sowie Schlafstörungen. Daher ist ein Verschluss des Septums angezeigt, um einen konstanten nasalen Luftstrom wiederherzustellen. Aktuell gibt es 2 therapeutische Strategien zur Behandlung von NSD-Symptomen: (1) den Einsatz von pflegenden Sprays und Salben zur Linderung der Beschwerden und (2) kausal der chirurgische bzw. nichtchirurgische Verschluss der Perforation. Die Perforation kann temporär mit einsetzbaren Nasenseptumbuttons oder operativ dauerhaft mit Eigengewebe verschlossen werden. Die Implantate werden anhand von analogen und digitalen Modellen gefertigt. Die Modelle werden entweder aus Silikonabformungen unter Vollnarkose oder in lokaler Betäubung oder aus computertomographischen(CT‑)Daten generiert. Beide Methoden haben jedoch einen Einfluss auf die Patientensicherheit, da sie das Morbiditätsrisiko erhöhen oder die Strahlenhygiene verletzen.

## Zielsetzung

Die vorliegende Arbeit befasst sich mit der nichtchirurgischen Behandlung von NSD mittels eines temporären Verschlusses. Dazu stellen die Autor:innen eine neue bildbasierte, kontakt- und strahlungsfreie Messmethode unter Verwendung eines Stereoendoskops vor. Es werden die 3D-Endoskop-Messergebnisse mit den CT-Daten als Goldstandard verglichen. Das Ergebnis dieser Arbeit soll einen Beitrag dazu liefern, inwiefern endoskopische/bildbasierte Messverfahren etablierte therapeutische Ansätze ersetzen/ergänzen können, um zukünftig das Patientenrisiko zu reduzieren.

## Klinische Beschreibung von NSD

Der schichtübergreifende Defekt der Nasenscheidewand (Abb. 1b) verändert die Geschwindigkeit und den Volumendurchfluss in der Nasenhöhle. Diese Faktoren sind bekanntermaßen entscheidend für nasale Funktionen wie Geruchssinn, Filtration, Erwärmung und Befeuchtung der eingeatmeten Luft [10, 26]. Posteriore und kleinere Perforationen verursachen aufgrund der weiterbestehenden befeuchtenden Wirkung der Nasenschleimhaut und der Nasenmuscheln tendenziell weniger Symptome [2, 23]. Die Prävalenz eines NSD liegt zwischen 0,9 und 2,1 % in der Bevölkerung und nach Septumplastik sogar bei bis zu 25 % [7, 13, 15, 24]. Es gibt dabei keinen bekannten Zusammenhang zwischen einem Septumdefekt und Faktoren wie Alter, Geschlecht oder geografischer Lage. Die Ursachen für NSD sind vielfältig; verschiedene Ätiologien wie traumatische Perforation, berufliche Exposition, persönliche Gewohnheiten, Drogenmissbrauch, topische oder systemische Medikation sowie bestimmte Autoimmunerkrankungen sind bekannt [4, 8, 16, 25, 28, 32].

## NSD-Therapie heute

**Figure 2:**
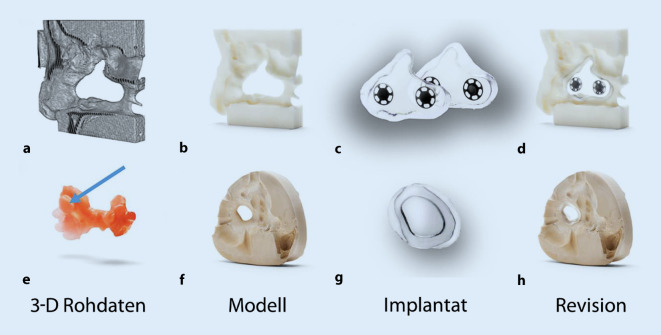

Grundsätzlich sollte ein NSD operativ verschlossen werden, wenn dessen Größe es zulässt und der Allgemeinzustand des Patienten nicht dagegen spricht [20, 21]. Eine Versorgung mittels industriell gefertigtem oder individuell angepasstem Button ist immer eine Kompromisslösung. Die nichtchirurgische NSD-Therapie durch temporären Verschluss erfolgt mit Implantaten, sog. Nasenscheidewandbuttons. Diese Implantate können einteilig sowie zweiteilig mit Magneten hergestellt werden (Abb. 2c, g). Aus den CT-Daten wird mittels einer Computer-Aided-Design(CAD)-Software ein 3D-Modell generiert (Abb. 2a). Aus den virtuellen Daten wird anschließend ein physisches Modell mit Computer-Aided Manufacturing (CAM) in einem 3D-Drucker hergestellt (Abb. 2b). Anhand dieses gedruckten Modells wird der zweiteilige Septumbutton mit Magneten hergestellt (Abb. 2c). Der fertige zweiteilige Septumbutton muss spannungsfrei und bündig am Modell anliegen (Abb. 2d). Analoge Abformungen (Abb. 2e) bieten eine weitere Möglichkeit zur Darstellung einer NSD. Aus diesen Abformungen wird ein Gipsmodell hergestellt (Abb. 2f). Aufgrund der Größenlimitierung der mit Silikon abzuformenden Perforation von etwa 3 cm^2^ wird i. d. R. ein einteiliges Implantat für den NSD hergestellt (Abb. 2g). In der Abb. 2h ist ein Septumbutton im analogen Gipsmodell dargestellt, auch hier müssen die Ränder spannungsfrei und bündig anliegen. Industriell konfektionierte Implantate sind nahezu obsolet aufgrund ihrer schlechten Passung und der damit verbundenen Krustenbildung. Diese kann unter den Kofaktoren lokale Infektion, Manipulationen durch den Patienten oder Arzt im Rahmen der Reinigung und durch Schleimhautnekrose aufgrund unzureichender Pflege zu einer Vergrößerung der Perforation führen. Zudem kann es bei ungenau passenden Buttons zu Druckstellen ebenfalls zu Schleimhautnekrose und konsekutiver Perforationsvergrößerung kommen. Der Septumbutton wird nach Abschwellung der Schleimhäute unter Anwendung eines topischen Lokalanästhetikums durch das Nasenloch in den entsprechenden NSD eingesetzt. Zweiteilige Septumbuttons werden mittels Magneten oder Druckknöpfen durch den Septumdefekt miteinander verbunden.

**Figure 3:**
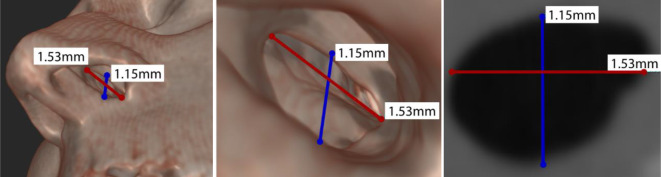

Alle Methoden haben jedoch Nachteile aus Patientensicht: Konfektionierte Implantate sind nicht patientenspezifisch und haben eine schlechte Passung, die zu Krustenbildungen führen kann. Bei sehr großen Defekten sind Silikonabformungen aufgrund fehlender Auflagepunkte größenlimitiert, außerdem erfordern sie eine Betäubung und können zu Verletzungen führen (erhöhtes Morbiditätsrisiko). CT-Daten stehen im Vergleich dazu im Konflikt mit der Strahlenhygiene. Jedes Verfahren erfordert unabhängig davon eine anatomische Vermessung der meist nicht kreisförmigen Perforation. Typische Perforationen können von wenigen Submillimetern bis zu mehreren Zentimetern reichen. Bei der Größenbestimmung ist es zudem wichtig, die vertikale als auch die horizontale Länge der Perforation zu messen (Abb. 3). Insbesondere die vertikale Perforationshöhe spielt eine Schlüsselrolle für den Therapieerfolg, da sie unmittelbare Auswirkungen auf die Spannung zwischen Nasenboden und Nasenrücken hat [4, 9]. Zudem ist eine spätere chirurgische Behandlung nicht ausgeschlossen. Für chirurgische Behandlungen sind mehrere Techniken mit unterschiedlichen Erfolgsraten beschrieben. Sie beruhen auf 2 Grundprinzipien:

- der Verwendung von Schleimhautlappen und
- dem Einsetzen eines Interpositionselements zwischen den beiden Schleimhautoberflächen [6, 14, 20–23].

Es muss jedoch immer eine Abwägung für die jeweilige therapeutische Methode erfolgen, da insbesondere Patienten mit hochgradigen Septumdeviationen unter aktiven Blutungen leiden können. Hier sehen die Autor:innen in der akuten Blutungssituation eine relative Kontraindikation zur Sofortversorgung. Darüber hinaus müssen bei Patienten mit aktiver lokaler Infektion oder unter Verwendung von intranasal zu applizierenden Medikamenten die Vor- und Nachteile der jeweiligen Versorgung kritisch überdacht werden [4, 12, 17].

## Endoskopische 3D-Vermessung in der Chirurgie

Bildbasierte strahlungsfreie 3D-Messverfahren wurden bereits in anderen chirurgischen Disziplinen verwendet, in denen die intraoperative Unterstützung von großem Interesse ist, u. a. in der robotergestützten Chirurgie, der 3D-Laparoskopie oder in der OP-Mikroskopie [1, 3, 11, 19, 30]. Im Vergleich zur Viszeralchirurgie und OP-Mikroskopie sind ein kleineres Sichtfeld sowie starke perspektivische Variationen durch extreme stereoskopische Blickwinkel die Hauptunterschiede für die Nasenchirurgie. Unter Berücksichtigung aller therapeutischen, technischen und chirurgischen Gegebenheiten besteht daher ein hoher Bedarf an der Entwicklung einer kontaktlosen und strahlungsfreien Messmethode zur Erstellung von patientenspezifischen anatomischen 3D-Implantaten bei gleichzeitiger Steigerung der Patientensicherheit und Implantatqualität.

## Methodik

**Figure 4:**
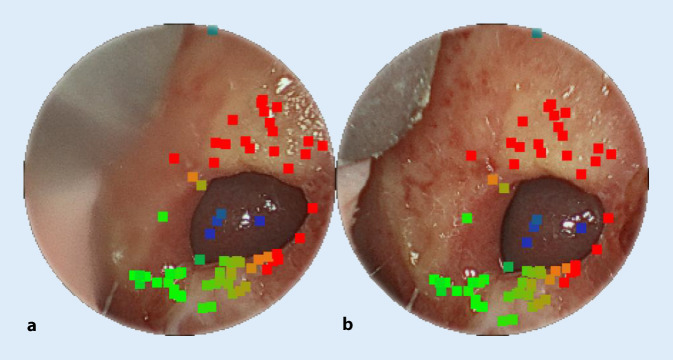

Die Autor:innen verwenden ein Stereoendoskop für die Aufnahme des NSD, um ein digitales 3D-Anatomiemodell zu berechnen. Dazu werden korrespondierende Merkmalspunkte bestimmt, die die gleichen anatomischen Landmarken für beide Stereoansichten beschreiben (Abb. 4). Daraus lässt sich die sog. Stereoparallaxe oder binokulare Disparität berechnen. Auf diese Weise können die Autor:innen bildbasierte Messungen mittels Triangulation bei bekannten Kameraeigenschaften (u. a. Brennweite) durchführen, um relevante 3D-Informationen aus den endoskopischen Bilddaten zu rekonstruieren.

### Studiengruppe

Diese Methode wurde mit der Zustimmung von 6 Patienten unterschiedlichen Geschlechts, Alters und mit unterschiedlichen Ursachen für den NSD evaluiert (Tab. 1). Die Studiengruppe bestand aus 3 jungen Erwachsenen (33–36 Jahre alt), 2 mittleren Alters (52–56 Jahre) und einer älteren Person (81 Jahre), die unterschiedliche Ätiologien und eine unterschiedliche Dauer des Septumdefekts seit der Erstdiagnose aufwiesen.

**Table 1:** 

Nr.	Geschlecht	Alter (Jahre)	Dauer (Monate)	Grund für Septumdefekt
*1*	Männlich	36	38	Drogenmissbrauch
*2*	Männlich	56	480	Spätfolgen/Komplikation Septumplastik
*3*	Weiblich	35	52	Granulomatose mit Polyangiitis/M. Wegener
*4*	Weiblich	52	84	Spätfolgen/Komplikation Septorhinoplastik
*5*	Männlich	81	264	Trauma
*6*	Männlich	33	26	Spätfolgen/Komplikation Septumplastik

### Bildgebung und Auswertung

Die Stereobilder wurden mit 2 verschiedenen Stereoendoskopen aufgenommen. Das erste Endoskop stammt von der Fa. XION MEDICAL GmbH, Berlin, und wurde speziell für HNO-Eingriffe konzipiert. Das zweite Endoskop ist ein 3D-Laparoskop der Fa. Schölly Fiberoptic GmbH, Denzlingen. Die Daten der Patienten Nr. 2, 3 und 5 wurden mit dem HNO-Endoskop (4 mm Durchmesser, 0°-Optik, 80° Öffnungswinkel, 60 fps („frames per second“, Bilder pro Sekunde), keine Zoommöglichkeit, Fokus veränderbar) aufgenommen, während die Daten der Patienten Nr. 1, 4 und 6 mit dem 3D-Laparoskop (10 mm, 0°-Optik, 72° Öffnungswinkel, 25 fps, keine Zoommöglichkeit, Fokus nicht veränderbar) aufgenommen wurden. Beide endoskopischen Systeme haben eine Bildauflösung von 1920 × 1080 px (Pixel) pro Stereokanal. Das Verfahren gliedert sich in mehrere Schritte und stützt sich auf echtzeitfähige Bildverarbeitungsalgorithmen [29]. Zunächst muss das 3D-Endoskop kalibriert werden, da die Messanwendungen und die Stereobildverarbeitung fest miteinander verknüpft sind. Die Kalibrierung eines optischen Bildverarbeitungssystems ist ein Offline-Vorverarbeitungsschritt, der die optischen Kameraparameter wie Brennweite, Stereobasis und Linsenverzerrungen berechnet. Für die Kalibrierdaten gilt, dass diese nur für eine feste Einstellung aus Zoom und Fokus Gültigkeit besitzen. Sollten die Vergrößerung oder die Fokuseben verändert werden, hat dies Einfluss auf die Messgenauigkeit. Dazu verwenden die Autor:innen eine geräteunabhängige Kalibrierung unter Einsatz eines schachbrettähnlichen Referenzkörpers [18, 31]. Darüber hinaus unterscheidet sich das hier eingesetzte Kalibrierverfahren von bekannten Methoden durch einen modellbasierten Ansatz mit Gradientenabstieg und Bildregistrierung zur Korrelation mit der Referenzebene [5]. Die komplette 3D-Rekonstruktions- und Vermessungskette besteht aus 3 Schritten:

- Stereorektifizierung,
- Stereoparallaxen‑/Disparitätsschätzung und
- die metrische Vermessung der Szene.

Die Rektifizierung des Stereobilds erfolgt über die Erkennung von robusten Merkmalspunkten ([33]; Abb. 4), um eine Homographie-Matrix abzuleiten, die garantiert, dass die Stereobilder frei von vertikalen Disparitäten sind. Anschließend wird eine Disparitätsschätzung [27] mit den korrigierten Stereobildpaaren durchgeführt. Die subpixelgenaue Disparitätsschätzung berücksichtigt zeitlich-räumliche Abhängigkeiten zwischen den zu bestimmenden Korrespondenzpunkten. Die Korrespondenzen werden dabei lokal ermittelt. Die iterative und unabhängige Verteilung der Korrespondenzen garantiert, dass die gesamte Szene unter Beibehaltung der zeitlich-räumlichen Konsistenz global aktualisiert wird. Nach einer kurzen Initialisierungsphase von 20 Stereobildpaaren erhält man eine komplette Repräsentation der Szene als Disparitätskarte. Abschließend rekonstruieren die Autor:innen die NSD-Anatomie maßstabsgetreu aus den berechneten Disparitätskarten und den vorab bestimmten Kamerakalibrierdaten. Auf den rekonstruierten Endoskopiedaten werden dann die horizontalen und vertikalen Dimensionen mittels Punkt-zu-Punkt-Messungen sowie der Umfang der Perforation direkt im Bild vermessen. Der Umfang wird durch die Akkumulation von mehreren Punkt-zu-Punkt-Messungen berechnet. Für den Vergleich erfolgte keine Registrierung der Messpunkte zu den CT-Daten. Die Vergleichsmessungen zwischen Endoskopiebildern und CT-Daten wurden hingegen an denselben anatomischen Stellen durchgeführt.

## Ergebnisse

**Figure 5:**
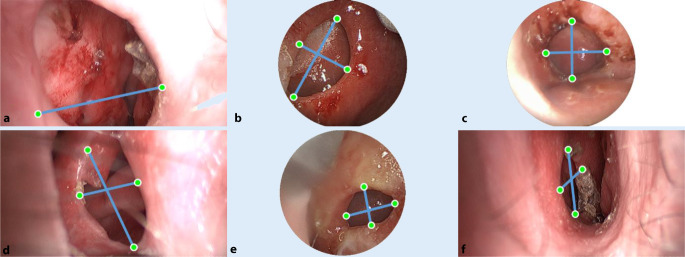

Die NSD aller 6 Patienten wurden mit einem der beschriebenen Endoskope aufgenommen. Die vorhandenen NSD sind in Abb. 5 mit ausgewählten Messpunkten dargestellt. In Tab. 2 gibt es einen Überblick über die bildbasierten Messergebnisse und einen Vergleich mit den bereits vorhandenen CT-Messungen. Die Schichtdicke der CT-Daten beträgt 0,5 mm bei Patient 1 und bei allen anderen 0,625 mm. Die Auswertung der dazugehörigen DICOM-Daten erfolgte mit der Software ImFusion Suite (ImFusion GmbH, München). Für alle Patienten mit Ausnahme von Nr. 1 wurde die axiale und koronale Achse sowie der Umfang der Perforation bestimmt. Die relativen Abweichungen liegen innerhalb der geforderten Messgenauigkeit von 5 %. Der Mittelwert aller Absolutdifferenzen beträgt 0,28 mm mit einer Standardabweichung (SD) von 0,146 mm für die axiale Achse und 0,16 mm mit einer SD von 0,09 mm für die koronale Achse sowie 0,904 mm mit einer SD von 0,769 mm für den Umfang. Bei allen durchgeführten Messungen betragen die Absolutfehler weniger als 0,5 mm und können somit vergleichbare Messwerte wie CT-Daten liefern. Es gibt keine signifikanten Unterschiede zwischen den beiden endoskopischen Systemen in Bezug auf Genauigkeit und Messunsicherheit. Die Messgenauigkeit bestimmt sich hier aus den Parametern des Stereosystems: Brennweite, Stereobasis, Bildauflösung, Pixelgröße sowie aus dem entferntesten Punkt, der in die Messung eingegangen ist. Patient Nr. 1 bedarf einer weiteren Erklärung (Abb. 5a), da die vorgeschlagene Methode keine sinnvolle axiale Messung und daher keine Bestimmung des Umfangs ermöglichte. Hierfür gibt es 2 Hauptgründe. Zum einem war es nicht möglich, die Perforationsausdehnung mit einer einzigen Aufnahme zu erfassen, da das endoskopische Sichtfeld aufgrund der extremen Größe der Perforation von 4,50 cm in der axialen Dimension überschritten wurde.

**Table 2:** 

	Stereoendoskop-Messung (mm)				CT-Messung (mm)				Relative Messabweichung (%)		
Nr.	Axial	Koronal	Umfang	Messunsicherheit	Axial	Koronal	Umfang	Voxelgröße	Axial	Koronal	Umfang
1	–	18,8	–	0,3	45,0	19,1	12,30	0,5	–	1,57	–
2	9,60	7,30	27,96	0,2	9,20	7,40	27,30	0,625	4,35	1,35	2,42
3	15,5	11,8	44,39	0,2	15,3	11,5	44,70	0,625	1,31	2,61	0,69
4	11,3	15,1	43,50	0,2	11,1	15,0	42,30	0,625	1,80	0,67	2,84
5	7,40	5,60	20,20	0,4	7,50	5,40	20,30	0,625	1,33	3,70	0,49
6	24,4	9,9	63,05	0,7	24,9	10,0	60,80	0,625	2,01	1,00	3,70

Außerdem konnten wichtige Bereiche aufgrund der stereoskopischen Verdeckung durch einen spitzen Blickwinkel nicht berechnet werden. Zum anderen wurde die Bildqualität durch die Bewegungsunschärfe der Kamera und durch atmungsbedingte Kondensation auf der Endoskopoptik negativ beeinflusst. Bewegungsunschärfe tritt bei beiden Objektiven auf, während Kondensation auch nur bei einem Objektiv auftreten kann. In beiden Fällen nimmt die Bildqualität stark ab, und es ist nicht mehr möglich, zuverlässige Korrespondenzen zu finden, um daraus die erforderlichen Tiefeninformationen zu berechnen.

**Figure 6:**
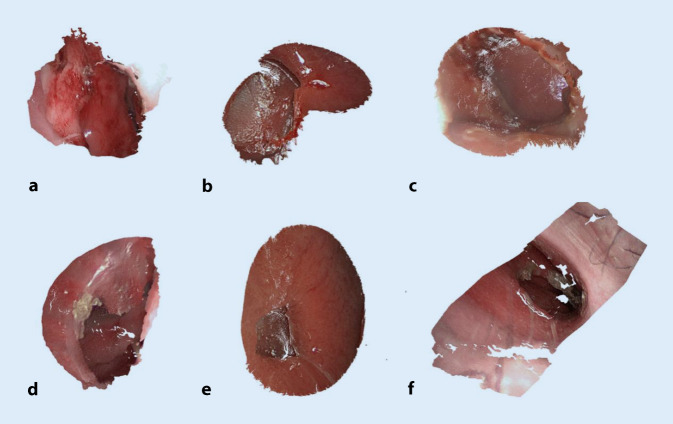

Neben den rein bildbasierten relevanten Vermessungsparametern wurden auch die dazugehörigen 3D-Punktwolken (Abb. 6) vollständig berechnet, um eine gezielte 3D-Visualisierung zur quantitativen Bewertung der Anatomie zu ermöglichen. Das hier eingesetzte bildbasierte 3D-Rekonstruktionsverfahren wurde bereits in der robotisch assistierten Chirurgie an 3D-Referenzanatomien von Schweinekadavern evaluiert. Es ist echtzeitfähig und liefert sehr genaue Messergebnisse mit sehr niedrigen Fehlerraten im Soll/Ist-Vergleich zur bekannten Referenzgröße [1]. Die hier erzielten Ergebnisse bestätigen somit die generelle Leistungsfähigkeit des 3D-Rekonstruktionsverfahrens für eine weitere chirurgische Anwendung mit zusätzlichen Anforderungen an die Algorithmik, die sich aus der speziellen Nasenanatomie sowie aus den stereosperspektivischen Besonderheiten ergeben.

## Diskussion und Ausblick

In der vorliegenden Arbeit haben die Autor:innen einen neuen bildbasierten Ansatz für die Vermessung von wichtigen Anatomieparametern bei NSD mit einem 3D-Endoskop vorgestellt und mit vorhandenen CT-Daten verglichen. Die Methode zeigt akkurate und präzise Messergebnisse mit geringen relativen Abweichungen im Submillimeterbereich zur Bestimmung der maßstabgetreuen Dimension des NSD in horizontaler und vertikaler Richtung sowie bei der Bestimmung des Perforationsumfangs. Bei der bildbasierten Methode kann der Schleimhautdefekt präzise erfasst werden, es erfolgt dabei im Gegensatz zur CT-basierten Methode aber keine optische Erfassung des Knorpeldefekts, welcher deutlich größer als der Schleimhautdefekt sein kann. Die Messungen erfolgen aktuell noch im 2‑D-Bild. Grafische CT-Vermessungen haben den Vorteil, dass vorliegende Septumdeviationen sowie individuell angepasste Buttons besser erfasst bzw. integriert werden können. Dies gilt auch für die Erfassung der Dicke des Septums am anterioren und posterioren Perforationsrand. Es konnten jedoch bereits jetzt hochauflösende 3D-Punktwolken erzeugt werden, die eine quantitative wie qualitative Beurteilung der Patientenanatomie durch die Chirurg:innen erlauben und zudem eine Erfassung der zuvor genannten Herausforderungen im Vergleich zu CT-Vermessungen möglich erscheinen lassen.

Nächste Schritte untersuchen die praktische Umsetzbarkeit dieses für den Patienten schonenden Ansatzes, um die 2 gängigen nichtchirurgischen Methoden (CT-Scan, Silikonabformung) zur Behandlung von NSD zu ersetzen. Unter anderem muss das Verfahren so erweitert werden, dass für alle Perforationsgrößen die entscheidenden NSD-Parameter vermessen werden können. Dies gilt insbesondere für NSD, die größer als etwa 3 cm^2^ sind und bei denen die chirurgische Szene nicht mit einer endoskopischen Einzelaufnahme erfasst werden kann. Die Therapie größerer Septumdefekte könnte mit dem hier erbrachten Nachweis von hochpräzisen Messergebnissen aus Einzelaufnahmen vielversprechend angegangen werden. Dazu sollen die Messungen und die Oberflächenrekonstruktion für beide Nasenlöcher zueinander mit sog. 3D-Mosaik- und SLAM-Verfahren („simultaneous localization and mapping“) zu einem globalen 3D-Modell registriert und fusioniert werden, sodass dieses für den 3D-Druck des Implantats genutzt und problemlos in die bestehenden Arbeitsabläufe von Epithetiker:innen integriert werden kann.

## Fazit für die Praxis

- Intraoperative bildbasierte Messverfahren mit 3D-Endoskopen erzielen vergleichbare Messwerte zu computertomographischen (CT-)Daten bei gleichzeitiger Vermeidung der Strahlenbelastung und sind beliebig oft im klinischem Verlauf wiederholbar.
- Endoskope sind – im Vergleich zu CT-Geräten – günstige Sensoren mit der Möglichkeit einer gezielten Erweiterung ihrer Funktionalität durch ergänzende Algorithmen der künstlichen Intelligenz (KI).
- Die 3D-Rekonstruktion aus endoskopischen Bilddaten bildet die Basis für zukünftige Augmented-Reality Anwendungen in allen chirurgischen Disziplinen.
- 3D-Rekonstruktionsverfahren bilden die generelle Basis für patientenspezifische Implantatlösungen in der HNO-Chirurgie.

## References

[1] Allan M, Mcleod J, Wang CC, Rosenthal JC, Fu KX, Zeffiro T, Xia W, Zhanshi Z, Luo H, Jia F, Zhang X, Li X, Sharan L, Kurmann T, Schmid S, Psychogyios D, Azizian M, Stoyanov D, Maier-Hein L, Speidel S (2021) Stereo correspondence and reconstruction of endoscopic data challenge https://arxiv.org/abs/2101.01133

[2] Bhattacharyya N (2007). Clinical symptomatology and paranasal sinus involvement with nasal septal perforation. Laryngoscope.

[3] Bodenstedt S, Wagner M, Mayer B, Stemmer K, Kenngott H, Müller-Stich BP, Dillmann R, Speidel S (2016). Image-based laparoscopic bowel measurement. Int J Comput Assist Radiol Surg.

[4] Downs BW, Sauder HM (2020). Septal perforation.

[5] Eisert P (2002). Model-based camera calibration using analysis by synthesis techniques.

[6] de Gabory L, Bareille R, Stoll D, Bordenave L, Fricain J-C (2010). Biphasic calcium phosphate to repair nasal septum: the first in vitro and in vivo study. Acta Biomater.

[7] Gold M, Boyack I, Caputo N, Pearlman A (2017). Imaging prevalence of nasal septal perforation in an urban population. Clin Imaging.

[8] Kridel RWH (1999). Septal perforation repair. Otolaryngol Clin North Am.

[9] Lee JY, Lee SH, Kim SC, Koh YW, Lee SW (2006). Usefulness of autologous cartilage and fibrin glue for the prevention of septal perforation during septal surgery: a preliminary report. Laryngoscope.

[10] Lindemann J, Keck T, Wiesmiller K, Sander B, Brambs H-J, Rettinger G, Pless D (2006). Nasal air temperature and airflow during respiration in numerical simulation based on multislice computed tomography scan. Am J Rhinol.

[11] Maier-Hein L, Groch A, Bartoli A, Bodenstedt S, Boissonnat G, Chang PL, Clancy NT, Elson DS, Haase S, Heim E, Hornegger J, Jannin P, Kenngott H, Kilgus T, Müller-Stich B, Oladokun D, Röhl S, dos Santos TR, Schlemmer HP, Seitel A, Speidel S, Wagner M, Stoyanov D (2014). Comparative validation of single-shot optical techniques for laparoscopic 3-D surface reconstruction. IEEE Trans Med Imaging.

[12] Neumann A, Schneider M, Tholen C, Minovi A (2010). Inoperable Nasenseptumdefekte. HNO.

[13] Oberg D, Åkerlund A, Johansson L, Bende M (2003). Prevalence of nasal septal perforation: the Skövde population-based study. Rhinology.

[14] Parry JR, Minton TJ, Suryadevara AC, Halliday D (2008). The use of fibrin glue for fixation of acellular human dermal allograft in septal perforation repair. Am J Otolaryngol.

[15] Peacock MR (1981). Sub-mucous resection of the nasal septum. J Laryngol Otol.

[16] Power DG, Kemeny NE (2011). Nasal septum perforation and bevacizumab. Med Oncol.

[17] Romo T, Sclafani AP, Falk AN, Toffel PH (1999). A graduated approach to the repair of nasal septal perforations. Plast Reconstr Surg.

[18] Rosenthal JC, Gard N, Eisert P (2017). Kalibrierung stereoskopischer Systeme für medizinische Messaufgaben.

[19] Rosenthal J-C, Gard N, Schneider A, Eisert P (2018). Microscopic image-based determination of stapes prosthesis length.

[20] Scheithauer M, Lindemann J, Sommer F, Wigand MCC (2021). Closure of nasal septal perforation. Laryngorhinootologie.

[21] Schultz-Coulon H-J (2005). Three-layer repair of nasoseptal defects. Otolaryngol Head Neck Surg.

[22] Susman E (2007) Fibrin glue makes septal perforations easier to repair. https://www.enttoday.org/article/fibrin-glue-makes-septal-perforations-easier-to-repair/. Zugegriffen: 24. Febr. 2020

[23] Tasca I, Compadretti GC (2006). Closure of nasal septal perforation via endonasal approach. Otolaryngol Head Neck Surg.

[24] Topal O, Celik SB, Erbek S, Erbek SS (2011). Risk of nasal septal perforation following septoplasty in patients with allergic rhinitis. Eur Arch Otorhinolaryngol.

[25] Traina TA, Norton L, Drucker K, Singh B (2006). Nasal septum perforation in a bevacizumab-treated patient with metastatic breast cancer. The Oncol.

[26] Uecker FC (2013). Hals-Nasen-Ohren-Heilkunde in Frage und Antwort.

[27] Waizenegger W, Feldmann I, Schreer O, Kauff P, Eisert P (2016). Real-time 3D body reconstruction for immersive TV.

[28] Watson D, Barkdull G (2009). Surgical management of the septal perforation. Otolaryngol Clin North Am.

[29] Wisotzky EL, Rosenthal J-C, Eisert P, Hilsmann A, Schmid F, Bauer M, Schneider A, Uecker FC (2019). Interactive and multimodal-based augmented reality for remote assistance using a digital surgical microscope.

[30] Wisotzky EL, Rosenthal J-C, Wege U, Hilsmann A, Eisert P, Uecker FC (2020). Surgical guidance for removal of cholesteatoma using a multispectral 3D-endoscope. Sensors.

[31] Zhang Z (1999). Flexible camera calibration by viewing a plane from unknown orientations.

[32] Zhao K, Dalton P (2007). The way the wind blows: implications of modeling nasal airflow. Curr Allergy Asthma Rep.

[33] Zilly F, Riechert C, Eisert P, Kauff P (2011). Semantic kernels binarized-a feature descriptor for fast and robust matching.

